# Supplementary material for: Global Pandemic Preparedness: Optimizing Our Capabilities and the Influenza Experience
Source: Vaccines (Basel). 2022 Apr 12;10(4):589. doi: 10.3390/vaccines10040589 (PMC9024617; doi:10.3390/vaccines10040589)
Supplement: Supplementary file 1 [file vaccines-10-00589-s001.zip › vaccines-1644537-supplementary.pdf]

Table S1. Established influenza vaccine manufacturers. Adapted with permission from Sparrow E, et al. Vaccine 2021;39:512-520 [1].

Manufacturer	Bulk vaccine production sites (countries)	Vaccine type	Source of publicly available information
Abbott Biologicals B.V	The Netherlands	IIV, egg-based	http://mri.cts-mrp.eu/download/NL_H_0137_001_FinalPL.pdf; https://www.abbott.com/corpnewsroom/finance/abbott-brings-its-proven-flu-fighters.html; https://www.medicines.org.uk/emc/files/pil.9381.pdf
Adimmune Corporation	China	IIV, egg-based	www.adimmune.com.tw/en/about_intro.php; www.adimmune.com.tw/en/service_products.php
AstraZeneca PLC	The United Kingdom	LAIV, egg-based	www.flumistquadrivalent.com/; www.azpicentral.com/flumistquadrivalent/flumistquadrivalent.pdf; https://www.fda.gov/media/139946/download
Bayerpaul Group	Iran (Islamic Republic of)	IIV, egg-based	www.bayerpaul.com/en-Pages-204/VACCINES
BIKEN Co., Ltd	Japan	IIV, egg-based	www.biken.or.jp/english/about_biken/products; www.pmda.go.jp/files/000153640.pdf
Changchun BCHT Biotechnology Co.	China	LAIV, egg-based	www.bchtpharm.com/100039/827.html
China National Biotec Group (CNBG)	China (2 facilities)	IIV, egg-based	https://www.cnbg.com.cn/; https://www.cnbg.com.cn/content/details_68_4440.html; https://www.cnbg.com.cn/content/details_68_380.html
CPL biologicals Pvt. Ltd	India	recombinant VLP, cell-based	http://cplbio.com/products/ http://cplbio.com/press-post/november-17-2016/
Daiichi Sankyo	Japan	IIV, egg-based for seasonal, cell-based for pandemic	www.daiichisankyo-bt.co.jp/business/index.html#s1; www.pmda.go.jp/files/000216875.pdf; https://www.daiichisankyo.com/media_investors/media_relations/press_releases/detail/006842.html

Manufacturer	Bulk vaccine production sites (countries)	Vaccine type	Source of publicly available information
Dalian Aleph Biomedical Co., Ltd.	China	IIV, egg-based	www.alephbio.com/Product/Content.aspx?ProductId=15 www.ncbi.nlm.nih.gov/pmc/articles/PMC4130289/
Denka Seiken Co., Ltd.	Japan	IIV, egg-based	www.denka.co.jp/eng/storage/news/pdf/203/20180709_flu_plant_eng.pdf; www.sciencedirect.com/science/article/pii/S2590136219300129; www.who.int/immunization/diseases/influenza/Table_clinical_evaluation_influenza_seasonal.xlsx
Fluart Innovative Vaccines Kft	Hungary	IIV, egg-based	http://fluart.hu/index.php/our-products/
FORT, ltd.	Russian Federation	IIV, egg-based	http://fort-bt.ru/; https://gmpnews.net/2018/07/fort-increases-its-sales-of-influenza-vaccine/; https://nacimbio.ru/en/vaccine/ultrix/; https://nacimbio.ru/en/vaccine/ultrix-quadri/
GC Pharma	Republic of Korea	IIV, egg-based	www.globalgreencross.com/eng/product/view.do?currentPage=1&idx=11&searchInitial=&searchValue=&searchColumn=&searchColumn2=1002&searchEffect=; www.globalgreencross.com/eng/product/view.do?currentPage=1&idx=22&searchInitial=&searchValue=&searchColumn=&searchColumn2=1002&searchEffect=
GlaxoSmithKline (GSK)	Canada, Germany	IIV, egg-based	https://us.gsk.com/en-us/about-us/vaccines/flu/; https://gsksource.com/pharma/content/dam/GlaxoSmithKline/US/en/Prescribing_Information/Fluarix_Quadrivalent/pdf/FLUARIX-QUADRIVALENT.PDF; https://gskpro.com/en-us/products/flulaval-quadrivalent/; https://www.pharmaceutical-technology.com/projects/gsk-biologicals/;

Manufacturer	Bulk vaccine production sites (countries)	Vaccine type	Source of publicly available information
			https://ca.gsk.com/media/1511156/gsk-at-a-glance_2018-update-28sept2018v2-final.pdf
Hualan Biological Engineering Inc.	China	IIV, egg-based	http://english.hualanbio.com/products/showproduct.php?lang=en&id=12; http://english.hualanbio.com/products/showproduct.php?lang=en&id=32
Il-Yang Pharm	Republic of Korea	IIV, egg-based	www.ilyang.co.kr/english/product/product01_view.asp?idx=555&page=1&txt_search=&sel_2nd_idx=&sl=&s_gubun=Vaccine
Instituto Butantan	Brazil	IIV, egg-based	http://butantan.gov.br/instituto-butantan/about-us; http://butantan.gov.br/instituto-butantan/main-products; https://www.ncbi.nlm.nih.gov/pmc/articles/PMC6069271/
Institute of Vaccines and Medical Biologicals (IVAC)	Viet Nam	IIV, egg-based	www.path.org/media-center/vietnam-produced-seasonal-influenza-vaccine-licensed-production-and-use/; http://ivac.com.vn/san-pham/10/41/ivacflu-s-(vac-xin-cum-mua-dang-manh-bat-hoat)/vien-vac-xin.html; http://ivac.com.vn/uploads/file/IVACFLU-S/HDSD%20CUM%20MUA%20V3%2014-6-19%20nam%20ban%20cau.pdf
Jiangsu GDK Biotechnology Co., Ltd.	China	IIV, egg-based	http://gdkbio.com/productshow.php@cid=3&id=28; https://pubmed.ncbi.nlm.nih.gov/28870140/; https://www.ncbi.nlm.nih.gov/pmc/articles/PMC7423295/
KM Biologics Co., Ltd.	Japan	IIV, egg-based for seasonal, cell-based for pandemic	www.kmbiologics.com/en/products/vaccines.html; www.pmda.go.jp/files/000213511.pdf; www.nature.com/articles/d42473-018-00213-x

Manufacturer	Bulk vaccine production sites (countries)	Vaccine type	Source of publicly available information
Mechnikov Institute	Nicaragua	IIV, egg-based	https://gmpnews.net/2019/04/launching-of-russian-vaccines-in-latin-america/; http://spbniivs.ru/projects_niivs/
Microgen	Russian Federation	IIV and LAIV, egg-based	www.microgen.ru/en/products/vaktsiny/vaktsina-dlya-profilaktiki-grippa-inaktivirovannaya-sovigripp/; www.microgen.ru/en/products/vaktsiny/ultravak-vaktsina-grippoznaya-allantoisnaya-zhivaya/
Sanofi Pasteur	China, France, Japan, Mexico, the USA (2 facilities)	IIV, egg-based and recombinant, cell-based	https://sanofiflu.com/fluzone-high-dose-influenza-vaccine.html; https://sanofiflu.com/fluzone-quadrivalent-influenza-vaccine.html; https://sanofiflu.com/flublok-quadrivalent-influenza-vaccine.html; www.sanofi.com/en/media-room/press-releases/2017/2017-10-12-07-00-00#:~:text=About%20Sanofi%20Pasteur,)%20and%20Shenzhen%20(China); www.biospace.com/article/sanofi-finally-closes-a-deal-pays-750-million-for-this-connecticut-biotech-/
Seqirus	Australia, the United Kingdom, the USA	IIV, egg-based and cell-based	www.seqirus.com/manufacturing/global-manufacturing-network; www.seqirus.com/products
Serum Institute of India Pvt. Ltd.	India	LAIV, egg-based	www.seruminstitute.com/product_influenza_vaccines.php; www.seruminstitute.com/health_faq_influenza.php; www.ciplamed.com/content/nasovac-s-intranasal-spray

Manufacturer	Bulk vaccine production sites (countries)	Vaccine type	Source of publicly available information
Sinovac Biotech Ltd.	China	IIV, egg-based	www.sinovac.com/?optionid=459
SK Bioscience	Republic of Korea	IIV, cell-based	www.skbioscience.co.kr/page/en/about02.do; www.skbioscience.co.kr/page/en/ourBusiness01.do
SPbNIIVS	Russian Federation	IIV, egg-based	http://spbniivs.ru/product/flu-m/; http://spbniivs.ru/wp-content/uploads/2019/11/flu_m_tetra.pdf
Takeda	Japan	Approved facility for cell-based pandemic IIV	www.takeda.com/newsroom/newsreleases/2014/takeda-selected-as-recipient-of-supplemental-government-subsidy-in-japan-to-expand-production-capacity-for-cell-culture-pandemic-influenza-vaccine/; www.takeda.com/newsroom/newsreleases/2014/takeda-announces-the-new-drug-application-approval-of-cell-cultured--influenza-vaccine-h5n1-takeda/
Zydus Cadila	India	IIV, egg based	https://zyduscadila.com/research; https://zyduscadila.com/public/pdf/pressrelease/Zydus-Cadila-launching-Tetravalent-Influenza-vaccine.pdf; www.hindustantimes.com/business/cadila-gets-nod-for-swine-flu-vaccine-launch/story-2erkQKbXiGJyzJtro79HkM.html

IIV, inactivated influenza vaccine; LAIV, live attenuated influenza vaccine.

1. Sparrow E, Wood JG, Chadwick C, Newall AT, Torvaldsen S, Moen A, Torelli G. Global production capacity of seasonal and pandemic influenza vaccines in 2019. Vaccine 2021;39:512-520. doi: 10.1016/j.vaccine.2020.12.018. PMID: 33341308.
